# Coronary Artery Calcification, Epicardial Fat Burden, and Cardiovascular Events in Chronic Obstructive Pulmonary Disease

**DOI:** 10.1371/journal.pone.0126613

**Published:** 2015-05-26

**Authors:** Thomas Gaisl, Christian Schlatzer, Esther I. Schwarz, Mathias Possner, Julia Stehli, Noriane A. Sievi, Christian F. Clarenbach, Damini Dey, Piotr J. Slomka, Philipp A. Kaufmann, Malcolm Kohler

**Affiliations:** 1 Department of Pulmonology, University Hospital Zurich, Zurich, Switzerland; 2 Department of Nuclear Medicine, University Hospital Zurich, Zurich, Switzerland; 3 Departments of Medicine and Biomedical Sciences, Cedars-Sinai Medical Center, Los Angeles, CA, United States of America; 4 Zurich Centre for Integrative Human Physiology, University of Zurich, Zurich, Switzerland; 5 Centre for Interdisciplinary Sleep Research, University of Zurich, Zurich, Switzerland; University of Dundee, UNITED KINGDOM

## Abstract

**Rationale:**

Patients with chronic obstructive pulmonary disease (COPD) suffer from significantly more cardiovascular comorbidity and mortality than would be anticipated from conventional risk factors. The aim of this study was to determine whether COPD patients have a higher coronary artery calcium score (CACS) and epicardial fat burden, compared to control subjects, and their association with cardiovascular events.

**Methods:**

From a registry of 1906 patients 81 patients with clinically diagnosed COPD were one-to-one matched to 81 non-COPD control subjects with a smoking history, according to their age, sex, and the number of classic cardiovascular risk factors (arterial hypertension, diabetes mellitus, dyslipidemia, family history of premature coronary artery disease). CACS, epicardial fat, and subsequent major adverse cardiovascular events (MACE) during follow-up were compared between groups.

**Results:**

Patients with COPD (Global Initiative for Chronic Obstructive Lung Disease-classification I: 5%, II: 23%, III: 16% and IV: 56%) showed no difference in CACS (median difference 68 Agatston Units [95% confidence interval -176.5 to 192.5], p=0.899) or epicardial fat volume (mean difference -0.5 cm^3^ [95% confidence interval -20.9 to 21.9], p=0.961) compared with controls. After a median follow-up of 42.6 months a higher incidence of MACE was observed in COPD patients (RR=2.80, p=0.016) compared with controls. Cox proportional hazard regression identified cardiac ischemias and CACS as independent predictors for MACE.

**Conclusion:**

COPD patients experienced a higher MACE incidence compared to controls despite no baseline differences in coronary calcification and epicardial fat burden. Other mechanisms such as undersupply of medication seem to account for an excess cardiovascular comorbidity in COPD patients.

## Introduction

Chronic obstructive pulmonary disease (COPD) is characterized by irreversible airflow limitation. Globally, COPD is the fourth leading cause of death in industrialized countries.[1] The clinical course of the disease is complicated by the development of systemic consequences and comorbidities, particularly atherosclerosis and coronary artery disease (CAD), which is one of the leading causes of death in COPD.[2] These comorbidities share common risk factors with COPD and possibly similar pathogenic mechanisms, but may also be a consequence of COPD itself and its pathophysiological effects on the vascular system.[3] The detection of new specific cardiovascular risk markers (e.g. atherosclerotic plaques in preliminary stages) is therefore crucial in this population in order to provide both powerful preventive and therapeutic measures.

Single-photon emission computed tomography (SPECT) is a robust diagnostic imaging tool which can assess previously unknown myocardial scar tissue or ischemia as potential predictors for future risk assessment.[4] Furthermore, the coronary artery calcium score (CACS) and the epicardial fat volume can be derived from the non-contrast computed tomography used for attenuation correction. The CACS is a well-accepted method for the detection of subclinical CAD at a very early disease-onset and acts as a strong predictor of future major adverse cardiovascular events (MACE).[5] Epicardial fat, on the other hand, is a relatively novel marker which reflects local adipose tissue within the visceral layer of the pericardium where it embeds the coronary arteries. Due to this proximity to the epicardial coronary arteries, it is thought that these fat depots may contribute to the development of coronary atherosclerosis through paracrine effects of inflammatory cytokines.[6] Furthermore clinical studies have suggested that epicardial fat is associated with vascular calcification[7] and may help to improve prediction of MACE[8] in asymptomatic patients. Therefore, CACS and epicardial fat may contribute additive prognostic information on the cardiovascular burden in COPD patients.

There are few and conflicting data on the relationship between COPD and subclinical CAD and some of the published studies are limited by inadequate matching of classic cardiovascular risk factors between COPD patients and control subjects.[9–12] The evidence to date warrants studies with follow-up and carefully considering the higher prevalence of hypertension, diabetes mellitus, and dyslipidemia among COPD patients compared with the general population.[13,14] Therefore, we conducted a one-to-one matched cohort study to evaluate whether COPD patients present a higher degree of coronary atherosclerosis and epicardial fat burden and to investigate the possible excess risk of COPD for MACE independent of established cardiovascular risk factors.

## Methods

### Study design

In this one-to-one matched cohort study COPD patients (at the University Hospital Zurich between 01.01.2007 and 31.07.2013) were screened for the following inclusion criteria: (1.) coronary artery calcium score (CACS) data available; (2.) history of smoking (smoker or ex-smoker with a smoking history of ≥10 pack-years [PY]); (3.) objectively confirmed COPD. COPD was staged according to Global Initiative for Chronic Obstructive Lung Disease [GOLD]-guidelines 2007[15] and spirometry results are expressed in percentage of predicted values according to the European reference equations[16]. Subjects were excluded according to the presence of one of the following exclusion criteria: (1.) coronary symptoms (Canadian Cardiovascular Society Angina Grading Scale ≥1) at the time of the SPECT investigation; (2.) known congenital or structural heart disease; (3.) previous heart transplantation. Controls were selected according to the same in- and exclusion criteria, however, without COPD. Classic cardiovascular risk factors were: treatment for (1.) hypertension, (2.) hypercholesterolemia, and (3.) diabetes mellitus, (4.) family history of premature MACE (first-degree family member: men <55, women <65 years of age), and (5.) obesity (body mass index [BMI] >30kg/m^2^). MACE were defined as documented death due to a cardiovascular event, non-fatal infarction (including stroke), documented transient ischemic attack, catheter-based target lesion revascularization, unstable angina pectoris requiring hospitalization, and coronary artery bypass graft.

Each COPD subject was 1-to-1 matched with one non-COPD subject for: (1.) sex (exact), (2.) number of classic cardiovascular risk factors (exact), and (3.) age. Age (normally distributed) was matched via nearest neighbour matching with a maximum allowable difference of one standard deviation between two participants. Matching was performed without replacement and optimal in order to minimize the total within pair differences. COPD subjects for whom no control case was found within the maximum absolute distance (n = 25) were left unmatched and excluded from analysis. Hence, only 81 (76%) of the COPD subjects were included in the analysis. Balance assessment in measured baseline covariates was additionally performed with the standardized difference, which is the difference in means in units of the pooled standard deviation.[17]

This study complies with the Declaration of Helsinki of the World Medical Association, ICH-GCP-Guidelines. The study protocol was approved by the Local Ethics Committee of the Canton of Zurich (KEK-ZH-Nr. 2013–0615) and registered with the study ID NCT02162095 (clinicaltrials.gov). Patients were included in the study if they had signed informed consent authorizing their records to be included in our cardiac imaging research registry. All patients underwent clinically indicated cardiac SPECT. Informed consent for the longitudinal part of the study (follow-up) was obtained separately from all patients or relatives in case of a deceased patient which was recorded by an investigator on the case report form. The ethics committee approved this procedure due to the low risk category this study entails.

### SPECT

All patients underwent a one-day pharmacological stress/rest SPECT Myocardial Perfusion Imaging protocol according to the guidelines of the European Association of Nuclear Medicine.[18] All cardiac SPECT studies were ECG-triggered. Data acquisition was performed with a dual-head detector hybrid SPECT/CT camera (Millenium VG and Hawkeye; GE Healthcare) or an ultrafast CZT camera (Discovery 530 NMc, GE Healthcare). Images were viewed on a dedicated workstation (Xeleris; GE Healthcare) and analysed by experts blinded to the grouping of patients.

The CACS was calculated in clinical routine using a commercially available semi-automatic software (SmartScore, GE Healthcare, Milwaukee, USA) according to the Agatston scoring method and expressed in Agatston Units (AU).[6] The area of coronary artery calcium was defined as automatically detected pixels with a noncontrast computed tomography density of ≥130 Hounsfield Units (HU) within manually selected lesions. The total CACS was computed, compromising the sum of the lesions in the left anterior descending artery, left circumflex artery, and right coronary artery. Epicardial fat and thoracic fat were calculated by two independent investigators (TG, MP) by a dedicated software (QFAT, Cedars-Sinai Medical Center, Los Angeles, CA, USA) which has been validated and previously described.[8,19,20] Conventional thresholds between -190 HU to -30 Hounsfield units (HU) were used to define three-dimensional fat voxels on a noncontrast computed tomography and findings are reported in cm^3^. Epicardial and thoracic fat were cranially confined by the first CT-slice displaying the bifurcation of the pulmonary trunk and caudally by the most inferior CT-slice of the myocardium. Epicardial fat was defined as detected fat-voxels within the visceral pericardium including all fat surrounding the coronary arteries while thoracic fat was defined as detected fat-voxels within the thoracic cavity limited by the posterior limit of the heart.[20] Therefore thoracic fat comprises epicardial fat. For each slice, pericardial contours were traced by manually placing a few control points (8–12) on the visceral pericardium, and epicardial fat, defined as fat voxels bounded by the visceral pericardium, was measured.[20] The thoracic fat was calculated automatically and corrected manually if necessary.[20]

### Follow-up

Follow-up was conducted either via personal contact, or with the help of the Swiss Federal Department of Justice and Police (Swiss register “Infostar”). Information was cross-checked and validated from internal and requested external medical charts with formal approval of the patient. All medical charts were obtained and MACE were critically reviewed by two investigators (TG, CS) unaware of the participants allocation. All deaths were categorized into cardiac or non-cardiac. The freedom from MACE time of a patient started at the time of SPECT imaging and ended at death/MACE, or at the last follow-up day. This method did not account for recurring MACE in one subject. Therefore, overall MACE rate was expressed as MACE/follow-up year.

### Statistical analysis

Continuous data are expressed as mean (±SD), or in the case of non-normal distribution by their median (quartiles). Paired tests (shared frailty) were used for matched pairs in this study. Ln(CACS+1) was used to investigate CACS-relationships with other variables. Continuous and categorical baseline characteristics of COPD and non-COPD subjects were compared using Wilcoxon and Pearson chi-square tests respectively. Spearman correlation analysis was used to investigate correlations with the CACS. Cumulative freedom from MACE at follow-up was analysed by means of the Kaplan—Meier method and compared between groups using the log-rank test. Univariable and multivariable (stepwise backward selection manner) Cox proportional hazard regression models were used to identify possible predictors of MACE. Furthermore, age, sex, and number of cardiovascular risk factors were also adjusted for multivariable analysis. Data analysis was conducted using IBM SPSS Statistics v. 22.0 software (IBM Corporation, Armonk, NY). A two-sided p value of <0.05 was considered to indicate statistical significance.

## Results

### Patient characteristics

**Fig 1:**
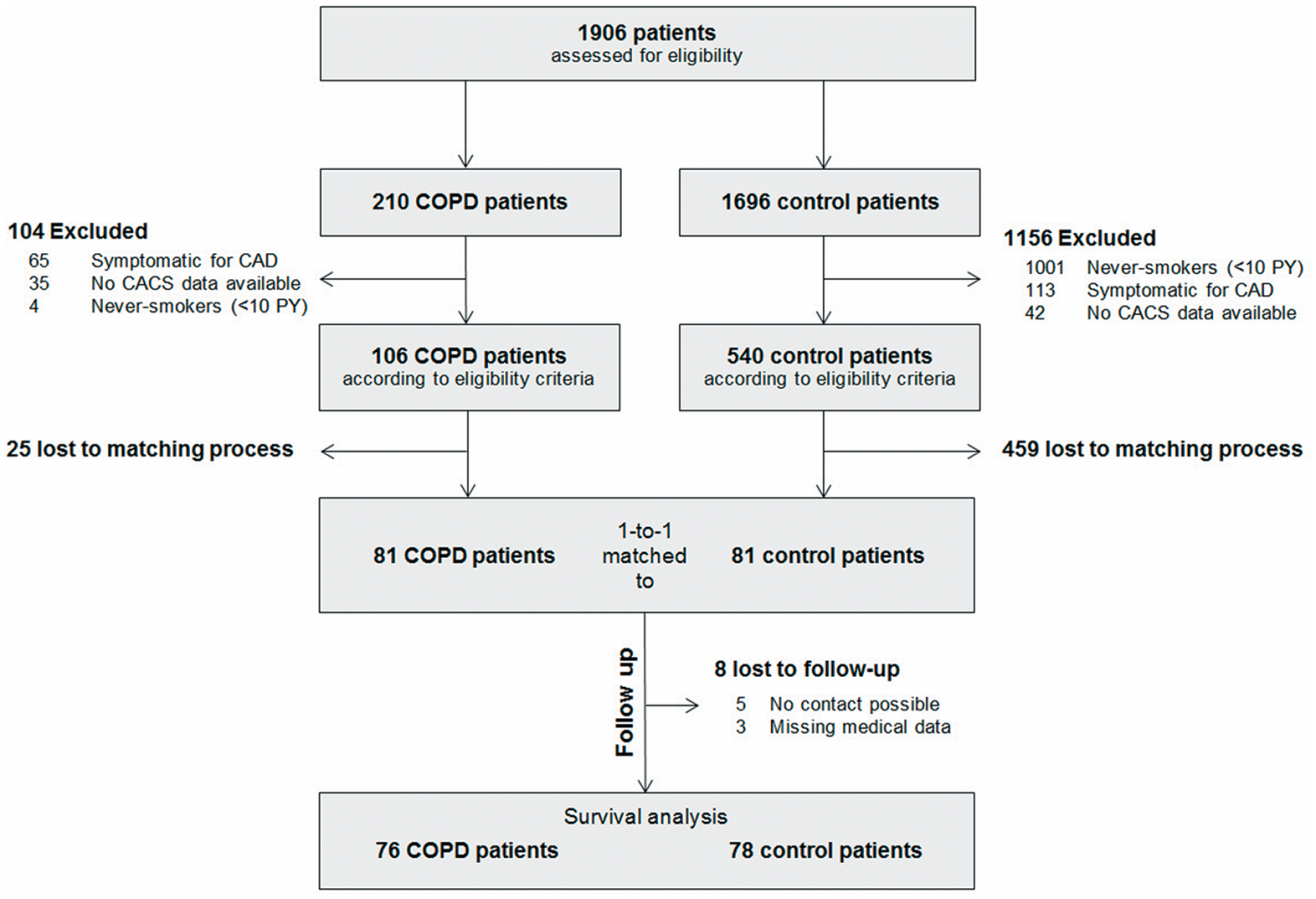
Patient flow chart.

Of the 1906 patients screened, 81 matched pairs entered the data analysis. Fig 1 summarizes the patient flow. The within-pair standardized age difference was 0.1%. Baseline characteristics are shown in Table 1.

**Table 1:** Patient characteristics.

	COPD group	Control group	p value
N	81	81	-
Age, years (±SD)	64.3 (±10.3)	64.3 (±9.1)	-
Sex (m / f)	63/18	63/18	-
Traditional cardiovascular risk factors, N (±SD)	1.4 (±1.1)	1.4 (±1.1)	-
Arterial hypertension, N (%)	51 (63)	50 (62)	0.782
Diabetes mellitus, N (%)	17 (21)	17 (21)	-
Dyslipidemia, N (%)	34 (42)	29 (36)	0.166
Family history for early cardiovascular events, N (%)	8 (10)	14 (17)	0.157
Obesity, N (%)	12 (15)	12 (15)	-
Body mass index, kg/m^2^ (±SD)	24.2 (±5.8)	26.7 (±4.9)	<0.001
CACS, AU (quartiles)	245 (38, 1404)	313 (47, 1061)	0.893
Epicardial fat, cm^3^ (±SD)	132.6 (±69.2)	132.3 (±90.2)	0.961
Thoracic fat, cm^3^ (±SD)	277 (±139.5)	283.0 (±149.8)	0.931
Left ventricular ejection fraction, % (±SD)	62.9 (±9.8)	63.47 (±10.7)	0.770
Scars in SPECT, N (%)	7 (9)	12 (15)	0.251
Ischemia in SPECT, N (%)	11 (14)	8 (10)	0.467
Current smoker, N (%)	25 (31)	36 (44)	0.071
CRP, ng/l^a^ (quartiles)	4 (1.1, 7.2)	4.1 (2, 10)	0.545
PY, N (±SD)	50.7 (±22.2)	31.7 (±20.0)	<0.001
FEV_1_ (% pred.)^b^ (quartiles)	28 (22, 66)	87 (68.8, 97.3)	<0.001
FVC (% pred.)^b^ (±SD)	71 (±19)	99 (±15)	<0.001
FEV_1_/FVC, %^b^ (quartiles)	37 (28, 58)	76 (71.8, 81)	<0.001
**GOLD Stage**			
I, N (%)	4 (5)		
II, N (%)	19 (23)		
III, N (%)	13 (16)		
IV, N (%)	45 (56)		
**Medication**			
ß-blocker, N (%)	20 (25)	40 (49)	0.001
Antihypertensive agents without ß-blocker, N (%)	51 (63)	59 (73)	0.059
Lipid-lowering agents, N (%)	28 (35)	29 (36)	0.617
Platelet aggregation inhibitor, N (%)	34 (42)	34 (42)	-
**Previous MACE**, N (%)	19 (23)	19 (23)	-
Stroke, N (%)	4 (5)	6 (7)	0.527
Myocardial infarction, N (%)	3 (4)	8 (10)	0.132
Coronary artery bypass grafting, N (%)	1 (1)	0 (0)	0.317
Percutaneous coronary intervention, N (%)	9 (11)	7 (9)	0.617

AU = Agatston units; CACS = coronary artery calcium score; COPD = chronic obstructive lung disease; CRP = C-reactive protein; FEV_1_ = forced expiration in one second; FVC = forced vital capacity; GOLD = Global Initiative for Obstructive Lung Disease; MACE = major adverse cardiovascular event; PY = pack years of smoking; SPECT = single-photon emission computerized tomography

^a^ Data available from 71 (87.7%) COPD subjects and 67 (82.7%) control subjects

^b^ Data available from 79 (97.5%) COPD subjects and 49 (60.5%) control subjects

The study population had a mean age of 64 years and was predominantly male (78%). All COPD GOLD stages were represented, but most of the COPD patients were in GOLD stage IV (I: 5%, II: 23%, III: 16% and IV: 56%). Apart from the anticipated differences in the lung function test and consumed PY, the two cohorts differed in BMI (but not obesity) and number of prescribed ß-blockers. Follow-up was successful in 154 (95%) patients, five patients were lost in the COPD group and three patients in the control group.

### SPECT

CACS did not differ significantly between the COPD group and the control group (245 [38, 1404] vs. 313 [47, 1061] AU, p = 0.893) nor in any GOLD stage compared to the controls adjusted for age, sex, number of cardiovascular risk factors, and smoking status (data not shown). Fat-quantification was successful in all but one patient (insufficient image quality). There was no significant mean (SD) group difference in epicardial and thoracic fat between the COPD group and the control group (132.6±69.2 vs. 132.3±90.2 cm^3^, p = 0.961 and 277±139.5 vs. 283.0±149.8 cm^3^, p = 0.931 respectively). Epicardial and thoracic fat correlated highly (r = 0.91, p<0.001) whereas correlation of epicardial and thoracic fat with CACS (r = 0.31, p<0.001 and r = 0.35, p<0.001 respectively) and BMI (r = 0.52, p<0.001 and r = 0.61, p<0.001) was moderate. CACS and epicardial fat correlated with the number of cardiovascular risk factors and detected scars in the SPECT (all p<0.001).

### Follow-up

**Fig 2:**
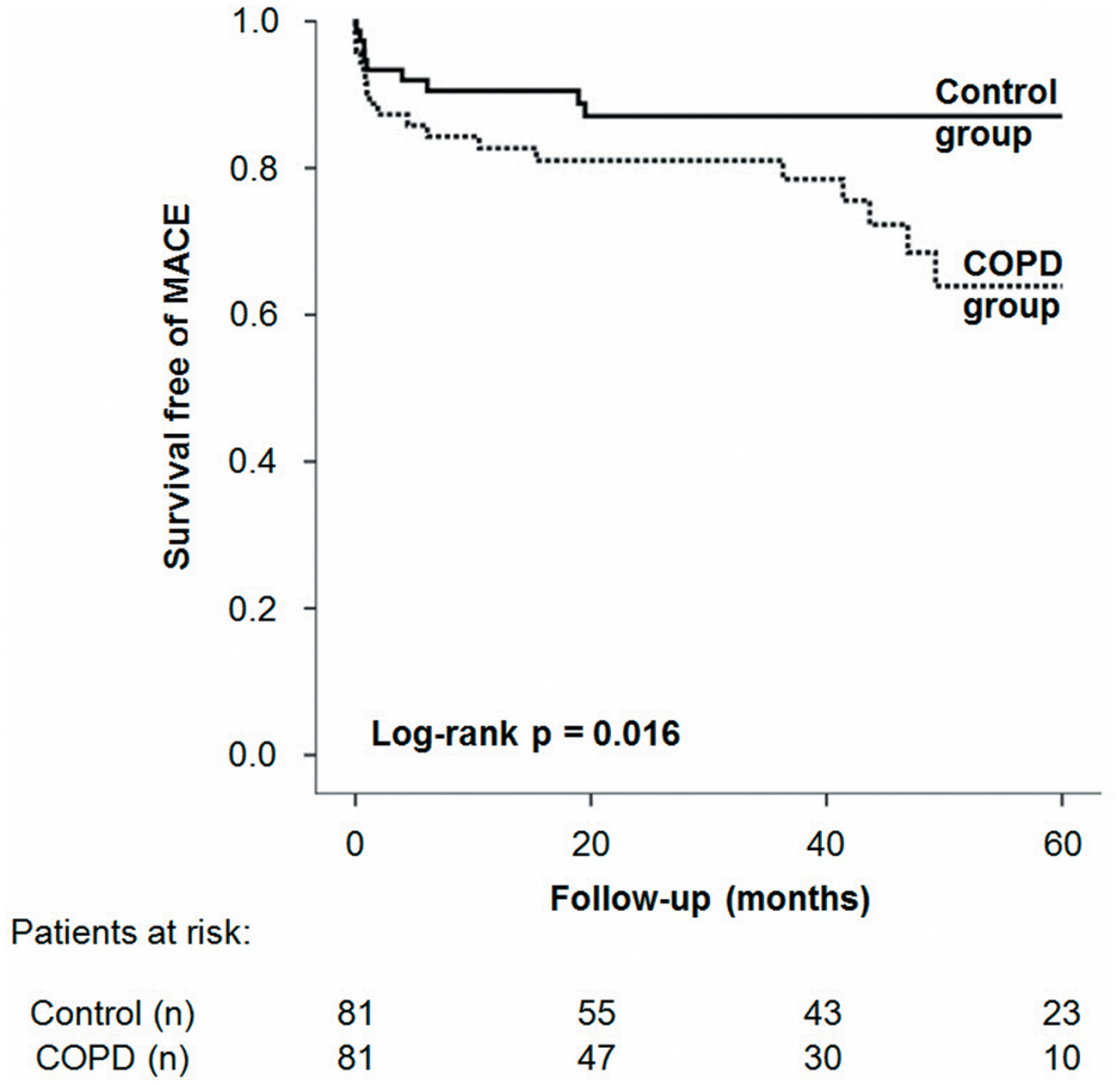
Kaplan-Meier curves for the study population.

During 410 person-years follow-up a total of 28 MACE (19 COPD vs. 9 controls, p = 0.030) and 48 deaths (26 COPD vs. 22 controls, p = 0.423) occurred. Median follow-up was 42.6 (21.1, 60.0) months and did not differ between groups (p = 0.543). Kaplan-Meier analysis (Fig 2) indicated that patients with COPD suffered from significantly more MACE (log-rank p = 0.016, RR = 2.80), especially myocardial infarction. The overall rate of death and myocardial infarction did not differ significantly between the COPD and control group (log-rank p = 0.115). MACE/follow-up year was 10.3% in the COPD group and 3.9% in the control group respectively (p = 0.015). CACS was significantly higher in patients with COPD who experienced MACE or death within the follow-up period compared to those who survived free of MACE and death (MACE: 1342 AU [387, 2414] vs. 204 [49, 1116] AU, p<0.001 / Death: 771 [46, 1659], vs. 218 AU [55, 1144], p = 0.011). For a summary on follow-up statistics see Table 2.

**Table 2:** Follow-up statistics.

	COPD group	Control group	p value
N	76	78	0.924
Lost to follow-up, N (%)	5 (7)	3 (4)	0.445
Median follow-up time, months (quartiles)	39.6 (20.8, 58.6)	46.0 (15.1, 60.0)	0.172
Patients with MACE, N (%)	19 (25)	9 (12)	0.030
MACE, N (%)	24 (32)	13 (17)	0.031
Annual MACE rate, %	10.3	3.9	0.015
Myocardial infarction, N (%)	5 (7)	0 (0)	0.022
Angina pectoris, N (%)	0 (0)	1 (1)	0.311
Percutaneous coronary intervention, N (%)	11 (14)	9 (12)	0.589
Stroke, N (%)	1 (1)	2 (3)	0.576
Transient ischemic attack, N (%)	3 (4)	0 (0)	0.077
Coronary artery bypass grafting, N (%)	2 (3)	1 (1)	0.538
Cardiac death, N (%)	2 (3)	0 (0)	0.153
Non-cardiac death, N (%)	26 (34)	22 (28)	0.423

COPD = chronic obstructive lung disease; MACE = major adverse cardiovascular event

Univariable logistic regression analysis demonstrated that COPD, CACS, FEV_1_ (% pred.), PY, ischemia in SPECT imaging, and previous CAD were risk factors for MACE (Table 3). In the multivariable Cox-regression analysis adjusted for age, sex, number of cardiovascular risk factors, BMI, ß-blockers, PY, and COPD, both ischemia in SPECT imaging and CACS were predictors of MACE (Table 4).

**Table 3:** Predictors of MACE in unadjusted analysis in a one-to-one matched cohort for age, sex and number of cardiovascular risk factors.

Predictors	Univariable B	Univariable 95% CI	p value
Age, (per 10 years)	1.00	1.00 / 1.00	0.744
Sex, male	1.62	0.65 / 7.18	0.211
Traditional cardiovascular risk factors, N	1.27	0.90 / 1.75	0.183
BMI, kg/m^2^	0.98	0.85 / 1.03	0.197
CACS, per 100 AU	1	1.00 / 1.00	0.002
Epicardial fat, cm^3^	1	0.99 / 1.00	0.973
Thoracic fat, cm^3^	1	0.99 / 1.00	0.371
GOLD classification	1.27	1.03 / 1.57	0.031
PY, N	1.01	1.00 / 1.03	0.033
Left ventricular ejection fraction, %	0.99	0.96 / 1.02	0.496
CRP, ng/l^a^	0.99	0.97 / 1.02	0.770
β-blocker	0.68	0.30 / 1.50	0.340
Initial ischemia in SPECT	3.32	1.45 / 8.22	0.005
Initial scar in SPECT	2.01	0.79 / 5.54	0.137
Previous coronary artery disease	2.35	1.15 / 5.37	0.020

AU = Agatston units; CACS = coronary artery calcium score; COPD = chronic obstructive lung disease; CRP = C-reactive protein; FEV_1_ = forced expiration in one second; FVC = forced vital capacity; GOLD = global initiative for chronic obstructive lung disease; MACE = major adverse cardiovascular event; PY = pack years of smoking; SPECT = single-photon emission computerized tomography

^a^ Data available from 71 (87.7%) COPD subjects and 67 (82.7%) control subjects

**Table 4:** Predictors of MACE in multivariable analysis adjusted for age, male sex, and number of cardiovascular risk factors.

Predictors	Multivariable B	Multivariable 95% CI	p value
CACS, per 100 AU	1.01	1.00 / 1.02	0.004
GOLD classification	1.13	0.85 / 1.50	0.389
Initial ischemia in SPECT	3.22	1.21 / 7.79	0.018
BMI, kg/m^2^	0.97	0.90 / 1.04	0.426
Pack years of smoking, yrs	1.02	0.99 / 1.03	0.073
Beta-blockers	0.75	0.28 / 1.98	0.564

AU = Agatston units; BMI = body mass index; CACS = coronary artery calcium score; GOLD = Global Initiative for Chronic Obstructive Lung Disease; MACE = major adverse cardiovascular event; SPECT = single-photon emission computerized tomography

## Discussion

This is the first one-to-one matched cohort study investigating whether COPD might be an independent risk factor for coronary atherosclerosis, epicardial fat burden and MACE. The main finding of this study is that although CACS and epicardial fat were not different in COPD patients compared to matched control subjects, MACE do occur more frequently in COPD and the incidence of MACE is independently associated with the degree of CACS. The underlying mechanisms between COPD and CAD are not fully understood. Mechanisms that have been proposed to explain the observed association between COPD and cardiovascular disease include systemic inflammation, hypoxia, oxidative stress, sympathetic activation, connective tissue alteration, increased platelet activation, impaired endothelial function and impaired physical activity.[3,21] COPD and CAD frequently occur concurrently, and the rate of non-fatal and fatal cardiovascular events is consistently increased in COPD patients.[22,23] The relationship between COPD and cardiovascular disease, particularly CAD, has been studied in previous observational cohort and case-control studies, and the degree of airflow limitation has been shown to be an independent predictor of cardiovascular events in the general population implying a causal relationship between airflow limitation and cardiovascular disease.[24] There is also evidence that CACS correlates with overall mortality in COPD patients.[25] Our data from this COPD cohort confirms the validity of the CACS in predicting MACE in this population whereas the association with mortality was non-significant. To date four studies investigating CACS as a surrogate for CAD in patients with COPD have been published which yielded inconsistent results.[9–12] One matched controlled study[12] found no difference whereas three studies using regression analysis found higher CACS in COPD patients when compared to non-COPD patients.[9–11] An explanation for some of the conflicting data could be the different study designs and/or deficiency of previous studies in controlling for potential cardiovascular (and atherosclerotic) risk factors which are highly prevalent in this population.[13,14] The correlation of the CACS with myocardial scars and other CAD risk factors in our study underpins the importance of matching. In respect of the study population, our study mainly included severe COPD cases, whereas the above mentioned studies mainly refer to COPD cohorts with mild or moderate disease. Furthermore, our findings are supported by the results of the large population based Multi-Ethnic Study of Atherosclerosis (MESA) study, which investigated 3642 participants aged 45–84 years without clinical cardiovascular disease and found no association between CACS and lung function or extent of lung emphysema.[26]

The Framingham heart study found an association of epicardial fat with coronary calcium and preliminary findings suggest epicardial fat may help to improve prediction of MACE.[7] While our results support the first association, epicardial fat was not associated with MACE in our study. Our data, however, show that epicardial fat is significantly associated with relevant CAD markers such as CACS, the number of cardiovascular risk factors, BMI, myocardial scars in SPECT, consumed PY, and male gender. To date, there is only one published study which investigated epicardial fat specifically in COPD patients.[12] In that study *Zagaceta et al*.[12] compared 171 COPD patients (with mostly mild disease) to 70 patients controlled for age, smoking history and BMI but nor for other classic cardiovascular risk factors and found that COPD patients had a higher epicardial fat volume (p = 0.02) than controls. Our study could not reproduce these findings in a COPD population with mostly severe COPD patients, as no association of COPD with epicardial or thoracic fat volume was found.

Perhaps the most intriguing finding of our study is the higher MACE incidence during follow-up in patients with COPD compared to the control group despite absent baseline differences in classic cardiovascular risk factors. This high MACE incidence is in agreement with results from a cohort study where a more than two-fold increase in the rate of hospitalizations due to cardiovascular events was found in patients with COPD compared with matched control subjects without COPD.[27] Underlying causes explaining the higher risk of cardiovascular events in COPD patients may include substandard medical care in regards to evidence-based medications, such as blood pressure medications. In the present study, patients with COPD were significantly less likely to receive antihypertensive agents, compared with the control group which is in line with further large population studies.[28,29] Possibly this is due to the fear, that especially non-selective ß-blockers and their assumed pulmonary side-effect in COPD patients may impair pulmonary function and increase the risk of disease exacerbation.[30] Thus it may be that substandard medical treatment in COPD patients is a contributor to the observed higher rate of MACE in such patients as several studies have demonstrated improved health outcomes are associated with ß-blockers in patients with COPD.[31]

A limitation of this study relies in the fact that CT-derived CACS is only a quantitative measure for overall calcium burden and therefore cannot assess morphological aspects of atherosclerotic lesions. A controlled coronary angiography study reported that COPD patients have worse morphological properties of atherosclerotic lesions which might explain the relatively high myocardial infarction-rates among COPD patients in our study.[32] The vulnerability of arteriosclerotic plaques is also thought to be influenced by the autonomic nervous system which has been shown to be altered in COPD patients.[33] Mechanisms like sympathetic overactivity and subsequent plaque rupture (due to hypoxia, inflammation, oxidative stress, physical inactivity and intrathoracic pressure changes)[33] but also impaired endothelial dysfunction[3] and subsequent vasospasm[34] have been suggested to contribute to the cardiovascular excess comorbidity in COPD patients. There is also epidemiological support for the concept that acute respiratory tract infections (which COPD patients are prone to) can precipitate MACE.[35]

Lastly, the possibility of lower levels of physical activity must be taken into account, as this is a frequently observed problem in patients with COPD.[36] The significance of exercise is somewhat supported by the findings of the recent NAVIGATOR trial which suggests that relatively small differences in daily physical activity (i.e. 2000 steps) may account for a higher risk of cardiovascular events in high risk populations.[37] However, in the current study we did not assess physical activity levels and thus this needs to be explored in future studies.

It is important to note that the study design of the current study does not allow establishing a causal relationship between COPD and cardiovascular excess comorbidity. However, it is extremely difficult to perform a randomized controlled trial to explore the cardiovascular effects of COPD as except for surgical treatment of emphysema (i.e. lung volume reduction surgery) there is no therapy which has a profound effect on disease severity. The relatively small sample size mainly due to restrictive 1-to-1 matching and limited amount of COPD patients undergoing SPECT also remains a limitation of this study.

In conclusion, there is an excess cardiovascular comorbidity in COPD patients which cannot be explained by coronary calcification or epicardial fat burden and thus further well designed studies with a focus on medication and comorbidities are needed to investigate the mechanisms underpinning the association between COPD and cardiovascular disease.
